# In-label, off-label prescription, efficacy and tolerability of dalbavancin: report from a National Registry

**DOI:** 10.1007/s15010-024-02176-2

**Published:** 2024-02-07

**Authors:** Silvano Esposito, Pasquale Pagliano, Giuseppe De Simone, Amedeo Guarino, Angelo Pan, Paola Brambilla, Claudio Mastroianni, Miriam Lichtner, Pierluigi Brugnaro, Anna Carretta, Teresa Santantonio, Gaetano Brindicci, Giuliana Carrega, Francesca Montagnani, Giuseppe Lapadula, Anna Spolti, Roberto Luzzati, Elisabetta Schiaroli, Vittoria Scaglione, Carlo Pallotto, Danilo Tacconi, Francesco Quintieri, Enrico Trecarichi, the Italian Society of Infectious, Tropical Diseases

**Affiliations:** 1 Department of Infectious Diseases, University of Salerno, Salerno, Italy; 2 Department of Public Health, University of Naples Federico II, Naples, Italy; 3 Department of Infectious Diseases, Istituti Ospitalieri of Cremona, Cremona, Italy; 4 Department of Public Health and Infectious Diseases, Sapienza University, Rome, Italy; 5 Department of Public Health and Infectious Diseases, Sapienza University, Latina, Italy; 6 Infectious Diseases Department, Ospedale Civile “SS. Giovanni E Paolo”, Venice, Italy; 7 Department of Infectious Diseases, University Hospital “Ospedali Riuniti” of Foggia, Foggia, Italy; 8 Department of Infectious Diseases, University of Bari, Bari, Italy; 9 Infectious Diseases Unit, Santa Maria Della Misericordia Hospital, Albenga, Savona Italy; 10 Department of Infectious Diseases, University of Siena, Siena, Italy; 11 Infectious Diseases Unit, San Gerardo Hospital, Monza, Italy; 12 Infectious Diseases Unit, Maggiore Hospital, Trieste, Italy; 13 Department of Infectious Diseases, University of Perugia, Perugia, Italy; 14 Infectious Diseases Unit, PO San Donato, Arezzo, Italy; 15 Infectious Diseases Unit, Magna Grecia University, Catanzaro, Italy

**Keywords:** In-label, Off-label, Dalbavancin

## Abstract

**Purpose:**

Although dalbavancin is currently approved for the treatment of ABSSIs, several studies suggest its efficacy and tolerance as long-term therapy for other off-label indications requiring prolonged intravenous antibiotic administration.

**Methods:**

We conducted a prospective nationwide study of dalbavancin use in real-life settings for both approved and off-label indications analysing for each case the clinical and microbiological characteristics of infection the efficacy and safety of treatments.

**Results:**

During the study period (from December 2018 to July 2021), the ID specialists from 14 different centres enrolled 223 patients treated with dalbavancin [141 males (63%) and 82 females (37%); male/female ratio 1.72; mean age 59 (SD 17.2) years, (range 15–96). Most patients in the study population (136/223; 61.0%) came from community rather than health care facilities and most of them were visited in Infectious Diseases wards (93/223; 41.7%) and clinics (55/223; 24.7%) even though some patients were cured in other settings, such as surgery wards (18/223; 8.1%), orthopaedic wards (11/223; 4.9%), Emergency Rooms (7/223; 3.1%) and non-surgical other than ID wards (6/223; 2.7%). The most common ID diagnoses were osteomyelitis (44 cases/223; 19.7%; of which 29 acute and 15 chronic osteomyelitis), cellulitis (28/223; 12.5%), cutaneous abscess (23/223; 10.3%), orthopaedic prosthesis-associated infection (22/223; 9.9%), surgical site infection (20/223; 9.0%) and septic arthritis (15/223; 6.7%).

**Conclusion:**

In conclusion, by virtue of its PK/PD properties, dalbavancin represents a valuable option to daily in-hospital intravenous or outpatient antimicrobial regimens also for off-label indications requiring a long-term treatment of Gram-positive infections.

## Introduction

Dalbavancin is a long-acting semisynthetic lipoglycopeptide discovered in 1996 from a fermentation product of the actinomyces *Nonomuria* spp. and approved by FDA in 2014 and EMA in 2015 for the treatment of Acute Bacterial Skin and Skin Structure Infections (ABSSSIs) [1].

The main characteristics of this molecule are its excellent activity against Gram-positive bacteria (including multidrug-resistant pathogens) and its long half-life (ranging from 149 to 250 h) allowing a once-weekly dosing regimen [2].

Resistance to dalbavancin is rarely reported and several studies demonstrated that dalbavancin efficacy and tolerance are non-inferior to vancomycin and other anti-Gram-positive molecules. Moreover, its extended half-life may ensure early discharge leading to lower risk of hospital-acquired infections and saving in public health; these advantages may therefore compensate for the cost of dalbavancin [3–5].

Based on preliminary investigations, dalbavancin can be considered a valuable choice in several settings, including off-label indications, such as infections sustained by Gram-positive multi-drug resistant (MDR) requiring prolonged intravenous antibiotic administration (such as endocarditis, blood-stream infections, osteomyelitis and prosthetic joint infections) allowing a reduction of the hospitalization period and relative costs [6, 7].

We conducted a nationwide analysis of dalbavancin use in real-life settings for both approved and off-label indications reporting for each case the clinical and microbiological characteristics of the infection and data on the scheme and setting of the antimicrobial therapy administrated.

## Patients and methods

Patients with a documented infection sustained by Gram-positive bacteria were included in this prospective study. During the study period (December 2018–July 2021), Infectious Diseases (ID) specialists from 14 ID Centres throughout Italy (5 in the Northern, 4 in Middle and 5 in Southern Italy) collected prospectively data concerning the use of dalbavancin, both for in-label and off-label indications, via an electronic case report form (eCRF).

The following information were collected for each patient and entered into the database: demographic data; co-morbidities; provenience (from community or healthcare facilities); the setting of visit (ID ward or clinic, surgery ward or clinic, emergency room); the infectious disease diagnosed; site and size of each lesion in case of SSTIs; presence of orthopaedic or vascular prostheses; results of microbiological exams, including the results of the susceptibility tests performed on the microorganisms isolated. The report form was completed by data regarding antibiotic treatments administrated before dalbavancin, including molecules administered, duration of therapies, route of administration and outcome. Setting of administration, duration, and side effects of dalbavancin and length of hospital stay was reported in each case. Failure of dalbavancin treatment was defined by lack of lesion healing or infection relapse despite appropriate management.

The Fisher exact test and a two-tailed X^2^ test were used to compare qualitative variables. Quantitative data were expressed as medians [interquartile range (IQR)] and compared using the Mann–Whitney U-test. Two-tailed P-values below 0.05 were statistically significant. The study was approved by the Ethical Committee (Ethical Committee Campania Sud approval 69/13–06-2018) for the Department of Infectious Diseases, University of Salerno, Italy, as principal investigator. The study was approved as well by the local Ethical Committees of each participating centre.

Before entering the study, each patient signed an informed consent to participate.

All information were collected according to current Italian legislation regarding the protection of privacy (D.L. No 196 30th June 2003).

## Results

During the 30-month study period, the ID specialists from 14 different centres collaborating to the study enrolled 223 patients treated with Dalbavancin [141 males (63%) and 82 females (37%); male: female ratio 1.72; mean age 59 (SD 17.2) years, (range 15–96)]. Indeed, in our population, the most represented age group was into the range 50–69 years (56 cases, 25%). No co-morbidities were reported in 75/223 cases (34%). Indeed, the most common comorbidities were cardiovascular diseases (52/223; 23%) and diabetes mellitus (35/223; 16%). Most patients in the study population (136/223; 61.0%) came from community and received dalbavancin in ID wards (93/223; 42%) and clinics (55/223; 25%). The other cases received dalbavancin in surgery wards (18/223; 8.1%), orthopaedic wards (11/223; 4.9%), Emergency Departments (7/223; 3.1%) and non-surgical other than ID wards (6/223; 2.7%).

**Fig. 1:**
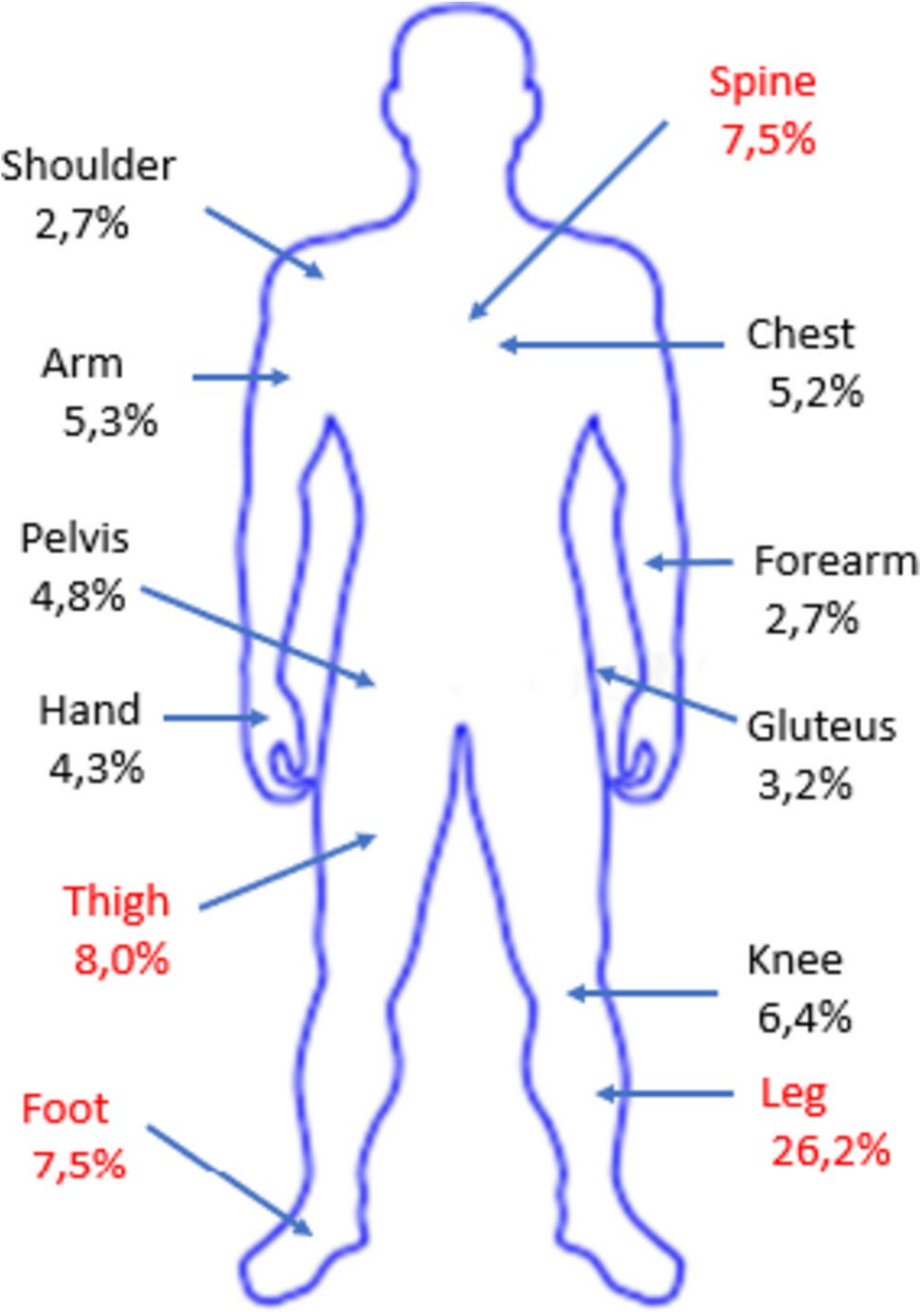
Anatomical sites of infectious diseases diagnosed

As expected, the most common ID diagnoses prompting dalbavancin administration were osteomyelitis (44 cases/223; 20%; of which 29 acute and 15 chronic osteomyelitis), cellulitis (28/223; 13%), cutaneous abscess (23/223; 10%), orthopaedic prosthesis-associated infection (22/223; 10%), surgical site infection (20/223; 9%) and septic arthritis (15/223; 7%), as reported in Table 1. Therefore, for 99 patients, dalbavancin was administrated off-label as summarized in Table 2. Most of the cases reported bone or skin and soft tissues lesions located were in the lower limbs (90/223; 40%). A spine infection was reported in 14 cases (8%). Endocarditis and CIED infections were reported in (Fig. 1).

**Table 1:** Diagnosis in the study population

Diagnosis	Number of cases out of total diagnosis (%)	
Acute osteomyelitis	29	13.0%
Cellulitis	28	12.5%
Abscess	23	10.3%
Prosthesis-associated infection	22	9.9%
Surgical site infection	20	9.0%
Septic arthritis	15	6.7%
Chronic osteomyelitis	15	6.7%
Erysipelas	14	6.3%
CIED-associated infection	11	4.9%
Wound infection	7	3.1%
Pressure ulcer infection	5	2.2%
Prosthetic valve endocarditis	4	1.8%
Native valve endocarditis	3	1.3%
Diabetic foot infection	2	0.9%
Other	25	11.2%
Total	223	100%

**Table 2:** Off label use of dalbavancin

Diagnosis	Number of cases (%)	
Acute osteomyelitis	29/223	13,0%
Prosthesis-associated infection	22/223	9,9%
Septic arthritis	15/223	6,7%
Chronic osteomyelitis	15/223	6,7%
CIED-associated infection	11/223	4,9%
Prosthetic valve endocarditis	4/223	1,8%
Native valve endocarditis	3/223	1,3%
**Total off label indications**	**99/223**	**44.4%**

The last line in bold reports the number of diagnosis treated with Dalbavancin as off-label

Microbiological exams were performed in 188 cases through the culture of several specimens depending on the type of infection as shown in Table 3: blood (58 cases; 31%), deep tissue biopsy (44 cases, 23%), biopsy (35 cases; 19%), superficial swab (21 cases; 11%) and prosthetic implant culture (9 cases; 5%). For 35 patients (16%), no microbiological investigation was done. Thirty-eight (20.2%) of 188 exams did not yield bacterial growth. Among the 150 patients reporting positive cultures, a monomicrobial infection was reported in 117 (78%) cases and a polymicrobial infection was reported in the remaining 33 (22%) cases (22%). Characteristics of the 167 bacterial isolates are reported in Table 4. As expected, the most common bacterial species found as causative agents in our study were represented by Gram-positive bacteria [139 cases, 83%)]. Gram-negative accounted for 11 cases (7%) reporting a polymicrobial infection. *Staphylococcus aureus* was detected in 85 (51%) cultures (51%) and methicillin-resistance rate was 47% (40/85). The second and the third most common aetiological pathogens were represented by coagulase-negative *Staphylococcus* spp. and *Staphylococcus epidermidis* with 26 (16%) and 16 (10%) cases, respectively, followed by *Enterococcus faecalis*, 9 cases (5%). Of the 223 patients in our study population, 35 were previously untreated with antibiotic therapy, the remaining 188 patients had been treated with other antibiotics and received dalbavancin because of previous treatment failure or as sequential therapy.

**Table 3:** Microbiological tests performed for diagnosis

Study population	223 patients
Patients with no microbiological exams performed	35/223 (15.7%)
Patients undergoing microbiological exams	188/223 (84.3%)
Microbiological exams performed	188 exams
Blood culture	58
Deep tissue	44
Biopsy	35
Superficial swab	21
Prosthesis	9
Drainage of secretions	4
Other	17

**Table 4:** Bacterial species isolated

Bacterial species	Number (%) of colonies from cultures	
Gram positives	139	(83.2%)
*Staphylococcus aureus*	85	(50.9%)
coagulase-negative *Staphylococci*	26	(15.6%)
*Staphylococcus epidermidis*	16	(9.6%)
*Enterococcus faecalis*	9	(5.4%)
Gram negatives	11	(6.6%)
*Pseudomonas* spp.	5	(2.9%)
*Escherichia coli*	2	(1.2%)
*Proteus* spp.	2	(1.2%)
*Acinetobacter* spp.	1	(0.6%)
*Klebsiella* spp	1	(0.6%)
Other	17	(10.2%)
Total species	167	

**Fig. 2:**
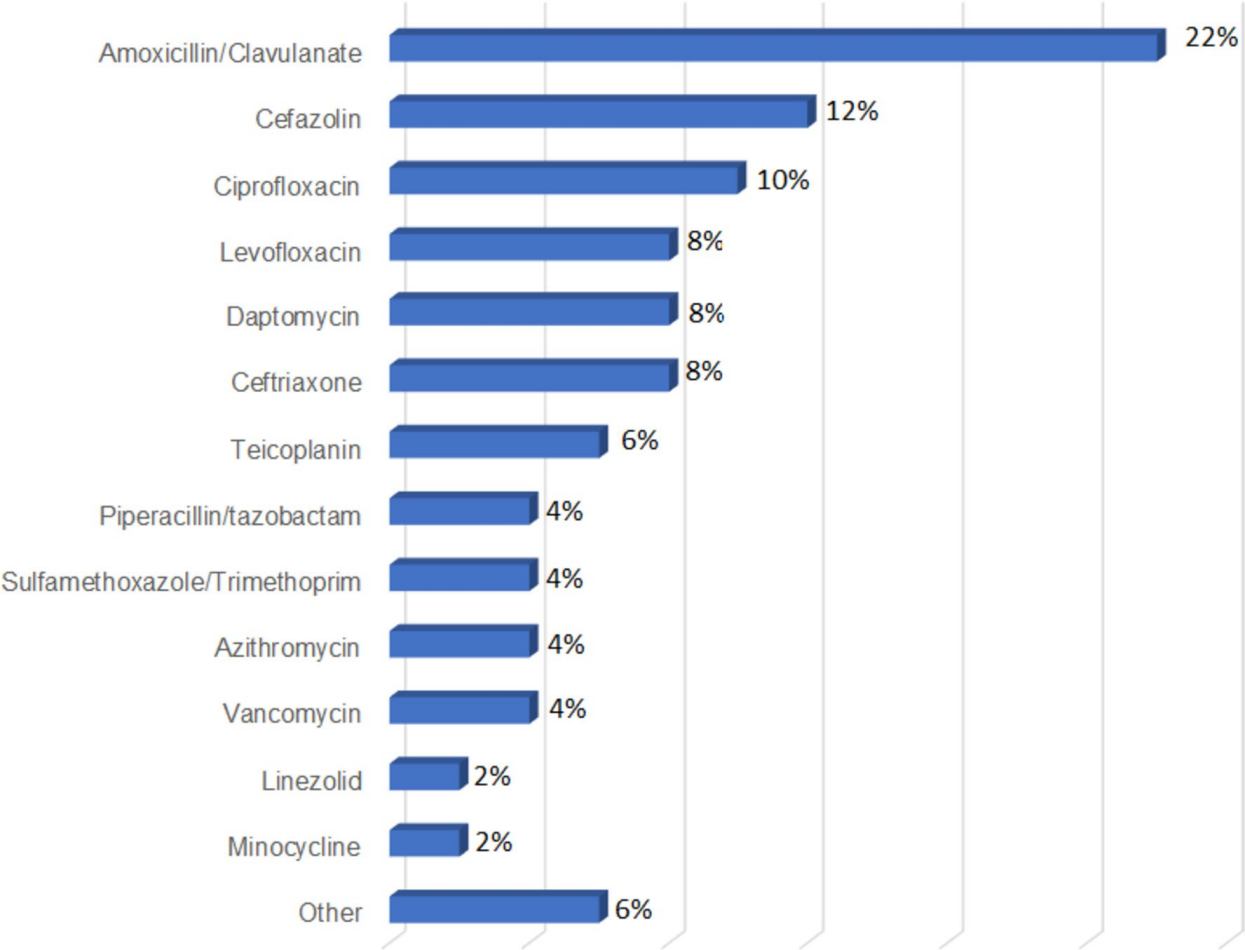
Previous antibiotic therapy administrated as mono-therapy

**Fig. 3:**
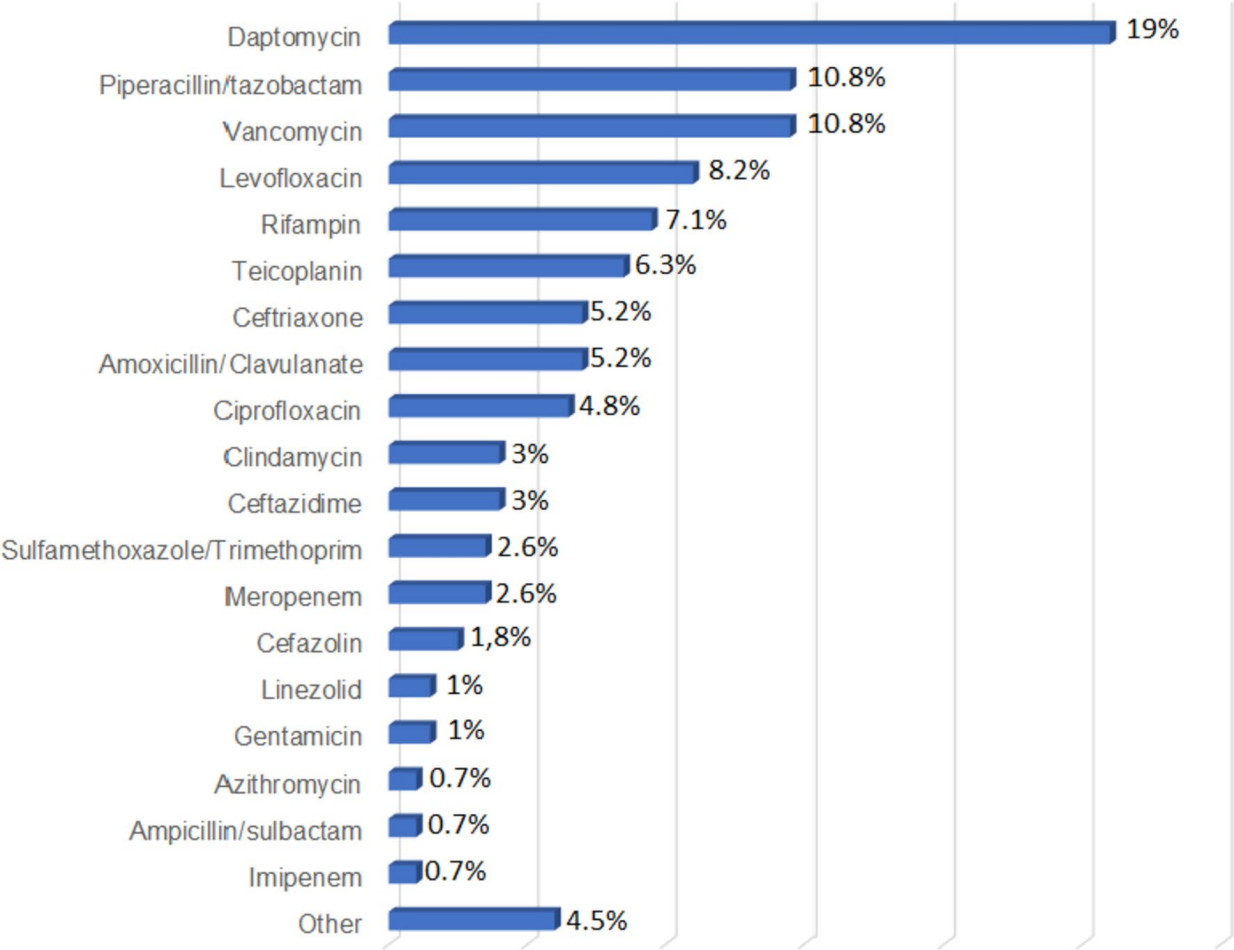
Previous antibiotics administrated as association-therapy

**Fig. 4:**
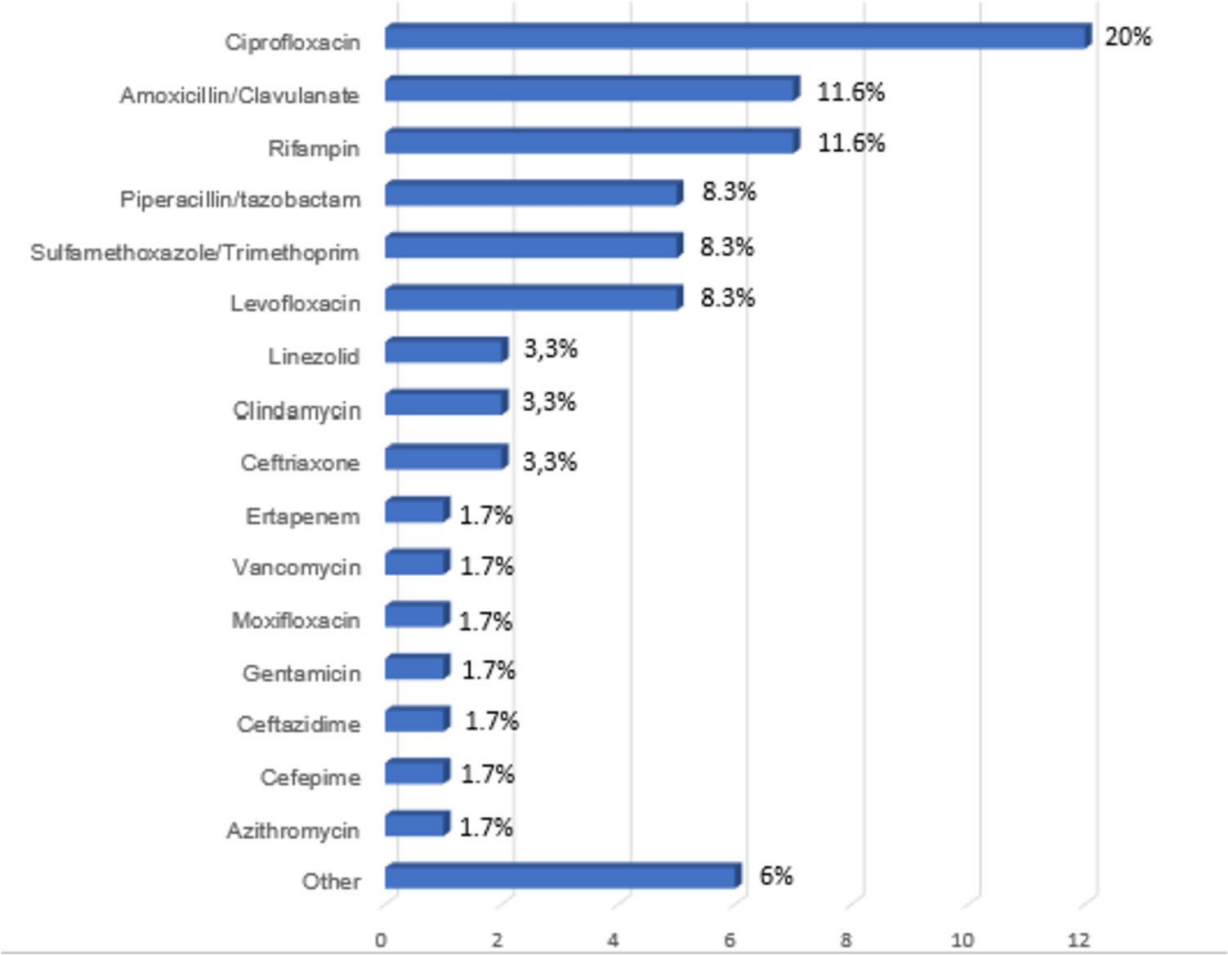
Antimicrobial molecules administrated in association with dalbavancin

**Fig. 5:**
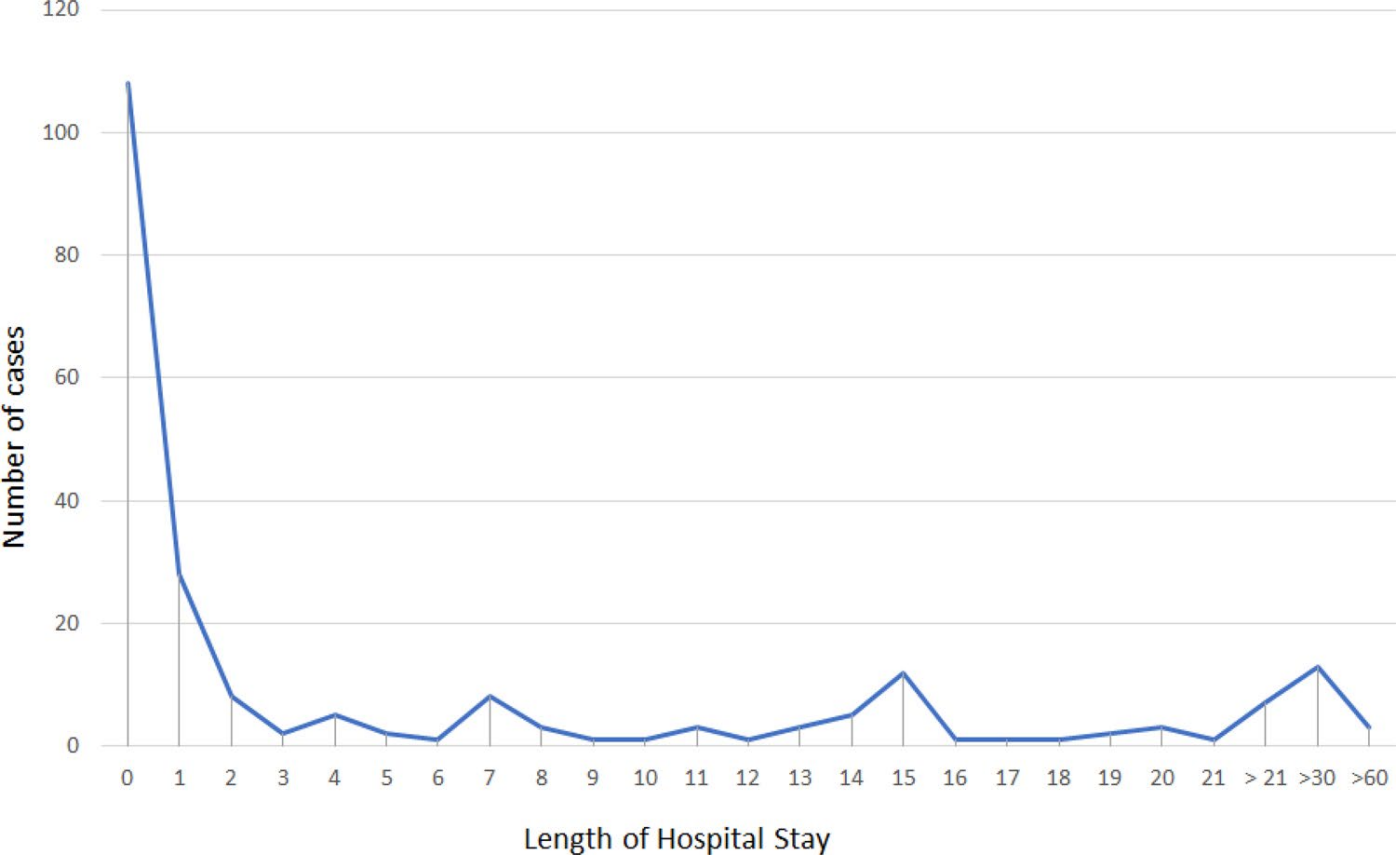
Length of hospital stay

In Figs. 2 and 3, we show the most frequent antibiotic regimens adopted, as monotherapy and as associative therapy, respectively, before the definitive treatment with dalbavancin. Comparing previous antibiotic treatments with those dalbavancin-based, we found that previous treatments had a lower duration (shorter than 7 days in 80%) and a higher failure rate (27% vs 1%), as reported in Table 5. In most cases, dalbavancin was administrated as monotherapy (163/223; 73%), while in 60 cases, dalbavancin was associated to other antimicrobial agents (27%) (Fig. 4). As regards the setting of administration, only 67 patients (30%) received dalbavancin infusion in hospital ward, in the remaining cases, dalbavancin was administrated in Day Hospital setting (109 patients; 49%) or in Infectious Diseases Clinics (47 patients; 21%). The mean length of hospital stay for those receiving dalbavancin in an hospital ward was 7 days, as reported in Fig. 5. The number of doses of dalbavancin administered ranged between 1 and 7, with a median number of 2 doses. Healing or improvement of the lesion was reached for 113 (51%) and 74 (33%) patients, respectively, while for nine cases (4%), no favourable result was observed and in nine (4%) cases, a relapse of infection was observed (non mi trovo con I conti). Only six patients (2.7%) experienced side effects: 4 patients had allergic rash, 1 patient reported nausea and 1 patient had joints pain; no side effects were reported in the remaining 217 (97.3%) cases. A sub-analysis on the patients for whom dalbavancin was used off label is reported in Table 6 and no statistical differences in outcome and adverse events was observed when compared to patients treated in label.

**Table 5:** Comparison between previous antibiotic treatment and Dalbavancin for all the infectious diseases diagnosed

		Previous treatment		Dalbavancin treatment	
		No	%	No	%
*Antibiotic therapy*					
	No therapy	35	15,5%	-	-
	Monotherapy	57	25,5	163	73%
	Association-therapy	131	59%	60	27%
*Route of administration*					
	Parenteral	134	71%	223	100%
	Oral	54	29%	-	
*Setting of administration*					
	Ward	126	67,5%	67	30%
	Domiciliary	55	29%	-	-
	Day Hospital	6	3	109	49%
	Clinic	1	0,5%	47	21%
*Duration of therapy (days)*					
	1–3	37	20%	20	9%
	4–7	43	23%	55	25%
	8–14	45	24%	107	48%
	15–21	27	14%	30	13%
	> 21	36	19%	11	5%
*Outcome*					
	Cure	-	-	123	(55%)
	Partial resolution	93	(49%)	82	(37%)
	Failure	81	(43,5%)	9	(4%)
	Relapse	14	(7,5%)	9	(4%)

**Table 6:** Comparison of off-label and in-label treatment of dalbavancin

		In-label dalbavancin		Off-label dalbavancin	
*Number of cases*		124	(55.6%)	99	(44.4%)
	Monotherapy	94	(75.8%)	69	(69.7%)
	Association-therapy	30	(24.2%)	30	(30.3%)
*Setting of administration*					
	Ward	47	(37.9%)	19	(19.2%)
	Day Hospital	43	(34.7%)	66	(66.6%)
	Clinic	34	(27.4%)	14	(14.1%)
*Doses administered*					
	1	102	(82.3%)	74	(74.2%)
	2	14	(11.3%)	17	(17.2%)
	3	5	(4%)	5	(5.3%)
	4	2	(1.6%)	3	(3.3%)
	> 4	1	(0.8%)	0	-
*Outcome*					
	Cure	62	(50%)	50	(50.5%)
	Partial resolution	45	(36.4%)	29	(29.3%)
	Failure	6	(4.8%)	3	(3.1%)
	Relapse	6	(4.8%)	3	(3.1%)
*Adverse events*					
	None	121	(97.6%)	96	(96.9%)
	Reported	3	(2.4%)	3	(3.1%)

## Discussion

Skin and soft tissue infections are the most common bacterial infections encountered both in ambulatory and hospital settings, but during the last 2 decades, they garnered even more attention, because their incidence is worryingly increasing worldwide, assuming the proportion of a global public health threat [8–13].

Both in health care facilities and in community setting, we are facing the increasing emergence of MDR microorganisms, which frequently needs expensive and long treatments via parenteral route and report a high failure rate [14]. In these settings, the administration of long-acting antimicrobials is an attracting therapeutic strategy, as it permits the administration of the treatments in ambulatory settings, avoiding hospitalization and finally reducing the burden of assistance associated to the presence of MDR bacteria in health-care settings [15–18].

Since its approval by FDA dalbavancin demonstrated its high efficacy and tolerability whenever utilized for its approved indication (ABSSSI), but over time, it became clear to clinicians that its pharmacokinetic/pharmacodynamic (PK/PD) properties could be used in other settings requiring long-term treatments, where its use allows a faster discharge from hospital and avoids the difficulties related to patient’s compliance to treatment [19–22]. Indeed, potential innovative therapeutic uses have emerged for dalbavancin which can be administered in many infections sustained by Gram positive resistant cocci such as osteomyelitis, prosthetic joint infections, endocarditis, bloodstream and vascular infections [23–25].

The present study, in contrast with the few other similar multicentre retrospective studies, was conducted as nationwide multicentre registry collecting prospectively information on dalbavancin use in real-life settings for both approved and off-label indications and reflects the current use of this drug.

During the study period, the ID specialists from 14 different centres collaborating to the study enrolled 223 patients treated with Dalbavancin, offering a considerable body of data about its efficacy and tolerability in many clinical settings. One-hundred-twenty-four (56%) patients received dalbavancin for ABSSSI treatment and 99 (44%) for an off-label diagnosis. The most common off-label diagnoses encountered were osteomyelitis, orthopaedic prosthesis-associated infection, endocarditis and CIED-associated infections, surgical site infection, and septic arthritis. In these cases, the cure rate was high, and the incidence of side-effects was low, suggesting that dalbavancin use in common practice can be successfully proposed in many settings where a long-term antibiotic treatment is required. Similar studies investigating the use of dalbavancin in the clinical practice have been conducted in different countries in the recent years and an increasing number of data are becoming available in the literature. Bouza et al. considered adult patients who received at least one dose of dalbavancin between 2016 and 2017 in 29 institutions in Spain. A total of 69 patients were treated (58% male; median age 63.5 years), being prosthetic joint infection (29%), acute bacterial skin and skin-structure infection (22%), osteomyelitis (17%) and catheter related bacteraemia (12%) the infections reported with the highest frequency. The authors highlighted that dalbavancin was used off-label in 79% of cases, reporting a high cure rate and a low incidence of side-effects [26]. Similar data are reported by another retrospective, observational, and multicentre study in Spain including 187 patients who received at least 1 dose between 2018 and 2019 in 7 Spanish hospitals. Osteoarticular (28%), cardiovascular (21%), and catheter-related infections (18%) constituted most cases receiving dalbavancin, confirming that a broader spectrum of infections can be successfully treated with dalbavancin [27].

Apart from those deriving from its efficacy comparable to the standard of care, main advantages deriving by dalbavancin use are associated with the potential cost savings, that are the reduction of the hospitalization period, which finally allows a faster return of the patients to its daily activities. This effect should be considered the highest in many off-label settings requiring parenteral administration of antibiotics for long periods [28].

Taylor et al. conducted in USA a retrospective, observational study conducted within a 4-hospital health system. Collecting information about adult patients who received dalbavancin from January 2018 to January 2021 for an off-label indication. Forty-eight patients met study criteria. Indications included osteomyelitis (54%), endocarditis (23%), bacteremia (15%), and prosthetic joint infection (8%) [29].

Lueking et al. conducted another observational study in USA including 40 patients treated with dalbavancin from February of 2019 to August of 2021. Indications for use included ABSSTIs (45%), bloodstream infection (67.5%), osteomyelitis (40%), infective endocarditis (10%), and septic arthritis (10%) [30].

Dinh et al. investigated over a 16-month period the first prescriptions of dalbavancin in France. Data from 75 patients from 29 French hospitals were collected via a standard questionnaire. The main indications were bone and joint infection (BJI) (64.0%), endocarditis (25.3%) as off-label diagnosis and SSTI as in-label diagnosis only in 17.3% of cases [31].

Other few studies available in literature concerning the off label use of dalbavancin were focused on specific diagnosis.

Ayka et al. in USA conducted a retrospective review of adult patients receiving at least one dose of dalbavancin between 1 November 2017 and 31 October 2019 for bacteremia or infective endocarditis, which typically could require outpatient parenteral antibiotic therapy (OPAT) for prolonged durations. At 90 days, eight patients (44%) achieved a clinical or biologic cure, six (33%) failed treatment, and four (22%) were lost to follow-up [32].

Bartoletti et al. conducted a retrospective, observational, cohort study of patients treated with dalbavancin for Deep Sternal Wound Infections over a 2-year period (March 2016 to April 2018) in two cardiac surgery departments in Italy. Fourteen patients received a first dose of 1000 mg followed by 500 mg, whereas 1 patient received 2 doses of 1500 mg each. All patients were defined as clinically cured. The median hospital LoS was 13 days (interquartile range, 8–18 days). At 6 months after discharge, 14 patients (93%) showed no relapse [33].

In our study, about 50% of the cases were treated with more than the scheduled two doses or received dalbavancin for an off-label diagnosis. Adopting these schedules, we reduced the costs related to the hospitalization for a difficult to heal skin lesion requiring a prolonged treatment period. Moreover, the same advantage in terms of savings of hospitalization could be highlighted in those receiving dalbavancin because of an off-label diagnosis. In fact, in these settings, dalbavancin can give several advantages including the reduction of the time needing for hospitalization of patients with life-threatening infections such as endocarditis or sepsis, that can receive the drug after the acute phase of the infection to complete the course of treatment scheduled for these cases. Indeed, the same advantage can be obtained in patients with prosthetic joint infection or osteomyelitis that should be treated for a long period, finally avoiding the threats related the patient compliance to such prolonged treatments. The results obtained by the analysis of our cases is confirmed by other similar studies. A study investigating patients with an ABSSSI highlighted cost savings approaching to 580 € per case treatment by transitioning from an inpatient to an outpatient setting, these costs derived by the reduction of the hospitalization period [34].

Savings deriving by dalbavancin administration can be the highest in patients experiencing wound infection after major surgery. A study comparing the costs related to the treatment of patients experiencing sternotomic wound infection with the standard of care (i.e. teicoplanin or daptomycin) or dalbavancin highlighted that cost saving approached to 16,000 € per case. The savings obtained accounted mainly to the reduction of the hospitalization period and did not impact on mortality [35].

Our study confirms together with all other studies available in the literature that dalbavancin has been used largely for off-label indication underlying that, whenever an infection prevalently sustained by Gram-positive cocci (more frequently methicillin sensitive or resistant Staphylococcus aureus) needs a long-term antibiotic treatment, dalbavancin represent a suitable option for its not only for its efficacy and safety but also) because offering a decreased lengths of stays and cost savings.

In conclusion, by virtue of its PK/PD properties, dalbavancin represents a valuable alternative to daily in-hospital intravenous or outpatient antimicrobial regimens in the treatment of long-term Gram-positive infections, in which hospitalization and employment of territorial medicine are strongly required.
